# Isotonic and Isometric Exercise Interventions Improve the Hamstring Muscles’ Strength and Flexibility: A Narrative Review

**DOI:** 10.3390/healthcare10050811

**Published:** 2022-04-27

**Authors:** Akhmad Fajri Widodo, Cheng-Wen Tien, Chien-Wei Chen, Shih-Chiung Lai

**Affiliations:** 1 International Sport Science Master’s Program, College of Human Development and Health, National Taipei University of Nursing and Health Sciences, Taipei 112303, Taiwan; fajriwidodo@gmail.com; 2 Physical Education Office, General Education Centre, National Taipei University of Nursing and Health Sciences, Taipei 112303, Taiwan; chengwen@ntunhs.edu.tw; 3 Department of Exercise and Health Science, College of Human Development and Health, National Taipei University of Nursing and Health Sciences, Taipei 112303, Taiwan; shihchiung@ntunhs.edu.tw

**Keywords:** athlete, exercise, injury, performance

## Abstract

Background: Hamstring weakness has been associated with an increased risk of hamstring strain, a common sports injury that occurs when athletes perform actions such as quick sprints. The hamstring complex comprises three distinct muscles: the long and short heads of the bicep femoris, the semimembranosus, and the semitendinosus. Methods: The researchers collected the data from different electronic databases: PubMed, Google Scholar, and the Web of Science. Results: Many studies have been conducted on the numerous benefits of hamstring strength, in terms of athletic performance and injury prevention. Isotonic and isometric exercises are commonly used to improve hamstring strength, with each exercise type having a unique effect on the hamstring muscles. Isotonic exercise improves the muscles’ strength, increasing their ability to resist any force, while isometric training increases strength and the muscles’ ability to produce power by changing the muscle length. Conclusions: These exercises, when performed at low intensity, but with high repetition, can be used by the healthy general population to prepare for training and daily exercise. This can improve hamstring muscle strength and flexibility, leading to enhanced performance and reduced injury risk.

## 1. Introduction

Hamstring strains are frequently observed in various athletes, from recreational to elite, and they typically occur during sports that require rapid sprinting and concurrent excessive muscle stretches, such as athletics and ball games [1,2,3,4]. Hamstring injuries are often slow to heal, and it is not uncommon for them to recur [5]. Nearly one-third of individuals who have a hamstring injury will reinjure themselves within a year of resuming their sport [6], and hamstring weakness has also been linked to an increased risk of hamstring strain [7].

Hamstring injuries are intricate and multifactorial in nature [8]. The proposed general risk factors for a hamstring injury can be classified into intrinsic (player-related) components and extrinsic (environment-related) factors [9]. Intrinsic risk factors for a hamstring injury include muscle weakness, instability, weariness, poor flexibility, poor technique, and psychosocial problems. Extrinsic risk factors include an insufficient warm-up period, training conditions, and playing surfaces [10]. Hamstring strength is also critical for knee joint stabilization, which directly affects the characteristics of agility manoeuvres, such as accelerations, decelerations, direction changes, and cutting [11].

Isotonic [12] and isometric exercises [13,14] are popular methods for developing hamstring strength. These interventions are frequently used as part of the physical preparation for a wide range of people, from injured members of the public to elite strength and power athletes. Changes that are frequently recorded following hamstring isotonic and isometric exercises include improvements in muscle mass, tendon quality, strength, power, range of motion, muscle fatigue delay, and voluntary activation [15].

## 2. Materials and Methods

The researchers collected the data from three different electronic databases: PubMed, Google Scholar, and the Web of Science. The data collected were limited to English language studies. Boolean logic (OR, AND) was used with keywords and other free terms to combine searches: (‘exercise’ OR ‘training’ OR ‘isotonic exercise’ OR ‘isometric exercise’) AND (‘muscle’ OR ‘hamstring’ OR ‘injury’ OR ‘biceps femoris’ OR ‘semitendinosus’ OR ‘semimembranosus’). The data collected in different databases followed their specific syntax rules. We also collected articles present in the reference lists of the resulting articles that were within the scope of this review.

## 3. Results

We selected 7 main articles out of 879 after deletion of the non-English literature. The articles are original studies that focus more on isotonic and isometric exercises (Table 1).

## 4. Discussion

### 4.1. Hamstring Structure and Function

Numerous studies have demonstrated a strong correlation between hamstring strength and athletic abilities, such as running and jumping [22,23]. The human hamstring muscle complex comprises three separate muscles: the semitendinosus, semimembranosus, and biceps femoris. This complex is involved in a wide variety of activities, from standing to explosive movements, such as sprinting and jumping [24]. Two of the muscles of the hamstring complex originate in the pelvis and run posteriorly along the length of the femur, via the femoroacetabular and tibiofemoral joints. The short head of the biceps femoris, in contrast, originates on the lateral lip of the femoral linea aspera, distal to the femoroacetabular joint [24]. As a result, some argue that it is not a true hamstring muscle. Unlike the biceps femoris, the other two hamstring muscles originate from the ischial tuberosity. The proximal long heads of the biceps femoris and semitendinosus muscles are connected by the aponeurosis. It extends roughly 7 cm from the ischial tuberosity. The distal hamstrings define the superolateral (biceps femoris) and superomedial (semimembranosus and semitendinosus) margins of the popliteal fossa. The gastrocnemius is primarily important for establishing the inferior border of the popliteal fossa [24].

The hamstring muscle complex is responsible for hip extension and knee flexion. It activates during the final 25% of the swing phase of the gait cycle, providing hip extension force and resisting knee extension. Additionally, the complex acts as a dynamic stabilizer of the knee joint. In conjunction with the anterior cruciate ligament (ACL), the muscles prevent the tibia from undergoing anterior translation relative to the femur during heel strike [25].

### 4.2. Muscle Types

The hamstring muscles are the three major muscles of the posterior aspect of the thigh. The semimembranosus is the medial-most muscle, the long and short heads of the biceps femoris are the lateral-most, and the semitendinosus is between them. This group of muscles is clinically significant, as it is highly susceptible to injury, especially in athletes [26].

Woods et al. described the anatomical distribution of hamstring injuries, observing that 53% of cases involved the biceps femoris, 16% the semitendinosus, and 13% the semimembranosus [27]. The biceps femoris is the strongest muscle in the hamstring complex, and it is responsible for knee flexion, external rotation, and posterolateral stability [28].

The biceps femoris muscle has both a long and short head, although the short head may be missing in some common variations. The long head originates from the medial ischial tuberosity and the inferior sacrotuberous ligament. The short head originates from the lateral lip of the femur’s linea aspera, the proximal two-thirds of the supracondylar line, and the lateral intermuscular septum. The two heads converge to produce the biceps femoris tendon, which is attached to the fibular head, crural fascia, and proximal tibia [28].

The semitendinosus muscle has been shown to exhibit stronger neuronal, metabolic, and stiffness responses than the other hamstring muscles during a knee flexion test, implying that the way in which the load is shared between these muscles results in a greater workload on the semitendinosus. However, because the hamstring heads differ physiologically and mechanically [29], the reactions to fatigue may also differ, in terms of active stiffness between the muscle components [30].

The semitendinosus is a solitary muscle, but it is more appropriately considered a digastric muscle physiologically, because of the presence of an intervening raphe into which the proximal fibres insert. These fibres arise from the upper region of the ischial tuberosity’s inferomedial impression via a tendon that connects to the long head of the biceps femoris. Of the three hamstring muscles, the semitendinosus has the shortest proximal tendon and the smallest physiological cross-sectional area (8.08 cm^2^) [31].

Caudal to the ischial tuberosity, the semitendinosus muscle becomes bulbous, with the semimembranosus tendon lying anterior to it. The semimembranosus muscle is frequently confused with the semitendinosus muscle, since the latter’s proximal tendon is not always a separate tissue. Further distally, the semitendinosus muscle produces a long tendon. This elongated distal tendon may exacerbate the muscle’s proneness to rupture [32].

Although the semimembranosus muscle may contribute to creating this conjoint tendon, this structure could be considered an anatomic variation [27]. The semimembranosus muscle originates on the superolateral part of the ischial tuberosity, beneath the proximal half of the semitendinosus muscle. The semimembranosus tendon runs between the medial and anterior sides of the other hamstring tendons. The proximal tendon is a lengthy structure that connects to the adductor magnus tendon and serves as the origin of the long head of the biceps femoris [25].

On the other hand, the semimembranosus, with its shorter fibre length, larger cross-sectional area, and shorter tendon, may contribute more to knee stabilization by stiffening the medial joint and resisting rotational loads. This selectivity of muscle activation within adjacent muscles has been demonstrated in studies of the lower and upper limbs using electromyography [33]. The semimembranosus is the largest muscle in the leg, with the longest proximal tendon. A broad aponeurosis joins the ischial tuberosity to its proximal insertion. The distal segment comprises tendinous branches joined to the popliteal fascia, the oblique popliteal ligament, and the posterior portion of the medial tibial condyle [34].

### 4.3. Injury

The majority of hamstring strains occur during intense sports, such as sprinting, which cause the muscles to overextend in response to abrupt changes in speed or direction. The most frequently damaged hamstring muscle starts from the biceps femoris, the semimembranosus to the semitendinosus [35]. Hamstring injuries typically cause posterior thigh pain, which is aggravated by knee flexion and hip extension. Patients may occasionally report hearing a “popping” sound with serious injuries. When clinicians evaluate a patient with a suspected hamstring injury, they must rule out other probable diagnoses, such as lumbosacral radiculopathy, adductor strain, or femoral stress fracture [2].

A grade 1 hamstring strain is defined as slight discomfort or swelling, little or minimal loss of function, absence of pain and functional impairment, and the presence of minor disruption of the hamstring myofibrils. A grade 2 hamstring strain is defined as recognizable partial tissue disruption with considerable pain, oedema, and function loss due to partial-thickness tears of the musculotendinous fibres. A grade 3 hamstring strain is defined as musculotendinous unit disruption or tear with severe discomfort, oedema, and loss of function [36].

Protection, rest, ice, compression, and elevation should be used to limit inflammation and swelling in the acute phase of hamstring injuries [37]. The patient’s pain threshold should regulate their range of motion, since excessive hamstring stretching may result in scar tissue formation [38]. The role of nonsteroidal anti-inflammatory drugs (NSAIDs) in treating hamstring injuries is debatable, with some studies indicating little benefit, while others indicate possible adverse effects [39]. However, short courses of NSAIDs (5–7 days) have been shown to not adversely affect recovery, but should be used purely as analgesics. Alternative pharmacologic therapies, such as platelet-rich plasma, have been found to assist recovery in athletes, although there are currently insufficient data to support the use of platelet-rich plasma to treat muscle strain injuries [40].

Fast and slow myosins could be markers of skeletal muscle injury [41]. The biceps femoris long head myosin heavy chain composition was not found to be ‘fast’, and, therefore, the composition does not appear to explain the high incidence of hamstring strain injury [42]. Furthermore, in vitro studies demonstrate that type II fibres (MHC-II) have a higher rate of force development [43] and greater maximum force at high velocities of shortening, yet the influence of MHC composition on in vivo hamstring function remains unknown.

Exercise programmes focused on eccentric contraction have been shown to significantly reduce the recovery time in patients who have healed sufficiently to resume therapeutic activities [24]. These regimens can be tailored to the patient’s recovery level and can contribute to reducing the injury recurrence rate [44]. Although hamstring stretching is widely recommended to reduce the risk of reinjury, there is no evidence that hamstring flexibility training reduces the rate of hamstring reinjury. Additionally, research has highlighted the crucial role of neuromuscular control in the lumbopelvic region. According to a 2004 prospective randomized research study, patients with acute hamstring strain injuries, who received rehabilitation using a progressive agility and trunk stabilization programme, had a reduced recurrence rate compared to those who received more typical progressive stretching and strengthening [45].

### 4.4. Isotonic Exercise

Isotonic exercise is a form of resistance training, and can be performed using free weights to strengthen a specific muscle area. Isotonic workouts emphasize comprehensive fitness, so they are popular with those looking to lose weight, improve muscle tone, increase physical power, or build their muscles. Examples of isotonic exercises include dumbbell curls and bench presses. However, isotonic exercise may result in delayed-onset muscular soreness [16].

In one study, a three-week regimen of isotonic hamstring exercise resulted in no improvement in pain or function [46]. Additionally, tendon pain may impair a patient’s ability to engage in sports, employment, and other daily activities. The effect of a hamstring injury on work and social activities is especially significant, since pain during sitting is often the predominant complaint [47,48]. Isotonic exercise has been well described in the literature, and it is clear from recent reviews that the greatest improvements in force and power production are observed at movement velocities similar to those used in training [49].

A typical isotonic exercise regimen is a standard gym programme, but with the weights moving at varying speeds. Throughout the workout, the approach emphasizes isotonic muscular contractions. A static weight is used to maintain muscle tension during the action. This affects the muscle, expanding the muscle cells and increasing the amount of weight that the muscle can lift. Alternatively, an isotonic exercise regimen is a form of muscular exercise intended to increase muscle strength and endurance. Since hamstring strains frequently occur during eccentric muscle contraction, stressing these muscles with eccentric exercise may help to avoid further hamstring strains [50].

The terms “concentric” and “eccentric” originate from basic physiology and refer to muscle contraction. Two types of muscular contractions have been described: isometric (no change in muscle length during contraction) and isotonic (constant tension in the contraction as the muscle length changes) [51]. Isotonic contractions are classified into two types: concentric and eccentric. Concentric contractions cause the muscular tension to increase in response to resistance, and then stabilize as the muscle shortens. Eccentric contraction causes the muscle to lengthen as the resistance exceeds the force produced by the muscle [51].

When the force applied to the muscle exceeds the instantaneous force produced by the muscle, eccentric (lengthening) muscular contraction occurs, resulting in the forced lengthening of the muscle–tendon system while the muscle contracts [52]. In comparison to concentric or isometric (constant-length) muscular contractions, eccentric muscle actions exhibit numerous distinguishing characteristics that may account for their unique adaptations [53]. Although they are not usually evident, eccentric muscular contractions are a necessary component of most motions performed during daily or athletic activities. Skeletal muscles contract eccentrically to support the body’s weight against gravity, absorb shock, and store elastic recoil energy for concentric (or accelerating) contractions [54].

The importance of such contractions in decelerating is revealed in downhill jogging or walking downstairs, which emphasizes the eccentric effort of the knee extensor muscles [55]. The favourable adaptive responses in hamstring strength and the architecture of the biceps femoris long head to eccentric hamstring exercise training are well documented [56].

Eccentric training has been found to elicit neuromuscular changes that are largely specific to each kind of muscle movement [57]. The eccentric role of muscles is critical in most sports. Eccentric muscular contractions enhance performance during the concentric phase of stretch-shortening cycles, which is critical for activities such as sprinting, jumping, and throwing. Muscles stimulated during lengthening motions can also operate as shock absorbers, assisting the athlete in decelerating during landing activities or accurately responding to strong external loading in sports such as alpine skiing [58]. Shorter muscle length has been shown to be a strong predictor of 100 m times in well-trained sprint runners [59], and increases in fascicle length have been observed in both a group of athletes who eliminated heavy resistance training and increased their high-speed training [60] and a group of resistance trainers who performed the concentric phase of their lifts at maximum velocity [60,61].

Isotonic exercise is effective for the development of hamstring muscle strength. Additionally, eccentric strength training has been shown to have a dramatic effect on overall hamstring muscle strength, with 29% and 19% improvement from eccentric and concentric exercise, respectively [62]. Both eccentric and concentric exercises have been shown to result in significant improvement in isotonic strength in the knee extensor muscles, which was related to hamstring strength, after a 12-week intervention [63]. In another study, hamstring stiffness increased significantly with isometric training (15.7%), suggesting that such training may be an important addition to ACL injury prevention programmes [13]. Eccentric hamstring exercises also help to improve some physical fitness components, such as the ability to sprint and jump over 5, 10, and 20 m and to countermovement jump [12].

Isotonic training consists of both eccentric and concentric exercises; thus, it positively affects the hamstring muscles, enabling them to produce more force and power, supporting performance. During eccentric and concentric muscle contraction, the muscle length changes, producing power. This training also affects the flexibility and stiffness of the muscles. Performing isotonic training may, thus, help to enhance the capacity of the hamstring muscles, to improve performance and reduce the risk of injury.

### 4.5. Isometric Exercise

Resistance training is used by a wide variety of people, from injured members of the general public to elite strength and power athletes [64]. Isometric training is gentle on the joints, while maintaining and increasing strength, making it ideal for individuals who require low-impact exercise due to an injury or arthritic condition. It also provides an excellent workout that is achievable without having to leave the house or go to the gym. Isometric exercises have recently been recommended to help alleviate and manage tendon pain [17].

Isometric exercises build muscle mass, strength, and bone density, while reducing cholesterol and improving digestion. Isometric contractions may, indeed, be more useful for improving muscles than eccentric contractions and flexibility [65]. Isometric exercise is, thus, a popular method of developing hamstring strength [12]. This exercise is a self-controlled bodyweight resistance variation of the traditional leg curl exercise. It does not require any equipment and provides an eccentric stimulus using bodyweight. Isometric training has been shown to alter physiological characteristics, such as muscle architecture, tendon stiffness and health [66], joint angle-specific torque [67], and metabolic activity [68]. Isometric contractions have been shown to carry a lower metabolic cost than concentric dynamic contractions. This leads to the hypothesis that performing maximal isometric contractions during warm-up may enhance subsequent explosive performance, while limiting the detrimental effects of accumulated fatigue [69]. Maximal isometric contractions during warm-up may also increase hamstring stiffness [13] because such stiffness is significantly correlated with isotonic muscle performance [70].

Numerous benefits have been attributed to isometric contraction training. Such training enables the application of force in rehabilitative settings to be firmly controlled within pain-free joint angles [71]. Isometric squat exercises that improve hamstring muscle contraction help to improve strength performance and kayaking sprints, relative to traditional exercises [72]. One study showed that isometric training resulted in improved hamstring isometric peak torque (9%), increased fascicle length (22%), and reduced pennation angle (−17%) in the biceps femoris long head [73]. Isometric training also enables the induction of force overload, since the maximal isometric force is larger than the force produced by concentric contractions [15]. Further, a practitioner who is familiar with the physical demands of a sport may be able to use isometric training to target specific weak areas in the range of motion, which can have a beneficial effect on performance [74] and injury prevention [9]. Finally, isometric contractions can also be used to provide analgesic relief and enable painless dynamic loading [17].

The previous study of isometric exercise suggested that a TheraBand (Hygenic Corp., Akron, OH, USA) was wrapped around the heel and the subject held the ends of the TheraBand in each hand [19]. The subject was instructed to keep the right knee locked in full extension and the hip in neutral internal and external rotation throughout the entire activity. The subject was then instructed to bring the right hip into full hip flexion by pulling on the TheraBand, attached to the foot, with both arms, making sure the knee remained locked in full extension at all times.

By performing a 70% maximal voluntary isometric contraction, study results show that a greater improvement in hamstring musculotendinous stiffness [13] and increasing the loading strategy with a heavy slow resistance program was helpful to the proximal hamstring tendinopathy [75].

Isometric exercises, thus, train the muscle to maintain its resistance ability against any force or under any conditions. They provide a simple and effective approach that can help to improve the hamstring muscles, and increase their resistance and power during activity. Most injuries to hamstrings occur because the muscles lack the strength to maintain the force or produce the amount of energy required.

## 5. Conclusions

The hamstring muscle group comprises the biceps femoris short and long heads, semitendinosus, and semimembranosus. Hamstring injuries are common in athletes of all levels, from beginners to professionals, and as is also explained, a myosin heavy chain does not appear to explain the high incidence of hamstring strain injury. Increasing hamstring muscle strength has traditionally been used as a preventative measure to reduce the risk of injury. Resistance training, consisting of both isotonic and isometric exercise, is widely used to treat hamstring muscle injuries. Isotonic exercise comprises both eccentric and concentric muscular contractions, and is defined as exercise that changes the muscle length, while isometric exercise results in no change in the muscle length. Isotonic training has been well described as the most effective method for increasing force and power generation, and isometric exercise improves hamstring muscle stiffness. While each of these exercise types has its own beneficial effects on hamstring muscle strength, judiciously combining the two may result in the greatest improvement in hamstring muscle strength for performance.

Applying both isotonic and isometric exercise may have different effects on the hamstring muscle. These exercises should be performed at low intensity and with high repetition by beginners, for 6–12 weeks. Once the muscle has adjusted to the exercise and requires a greater load to improve further, the load can be increased, and the volume or repetition of the exercise can be decreased. Most athletes use this type of exercise during the general preparation phase of their training, but the general population can use this exercise as well, depending on their needs. Muscle training is important for improving hamstring muscle capacity and reducing injury risk. Isometric exercise will train the muscles to maintain their ability to resist any force or under any conditions. Isometric exercise is a simple and effective approach to improve the resistance and power of the hamstring muscles during activity. Most hamstring injuries occur because the muscles lack the strength to produce the force or energy required.

## Figures and Tables

**Table 1:** Summary of related studies.

Authors	Year	Result
Rich A., Cook J.L., Hahne A.J., et al. [16]	2021	Isotonic (eccentric) exercise helps to increase hamstring muscle strength.
Rio E., Kidgell D., Purdam C., Gaida J., Moseley G.L., Pearce A.J., Cook J. [17]	2015	Quadriceps strength on maximal voluntary isometric contraction (MVIC) increased significantly following the isometric condition.
Seagrave, et al. [18]	2014	Nordic hamstring exercises may decrease the incidence or acute hamstring injuries by increasing the strength.
Nelson, R.T., Bandy, W.D. [19]	2004	Eccentric exercise helps to improve hamstring muscle flexibility.
Delahunt E., McGroarty M., De Vito G., Ditroilo M. [20]	2016	Improvement in hamstring muscle control during eccentric contractions.
Marusic J., Vatovec R., Markovic G., Sarabon N. [21]	2020	Short-term eccentric hamstring strengthening at long muscle length can have significant favorable effects on various architectural and functional characteristics of the hamstrings.
Van der Horst N., Smits D.W., Petersen J., Goedhart E.A., Backx F.J. [17]	2015	Nordic hamstring exercise protocol in regular amateur training significantly reduces hamstring injury incidence.
